# RNA-seq reveals a novel porcine lncRNA MPHOSPH9-OT1 induces CXCL8/IL-8 expression in ETEC infected IPEC-J2 cells

**DOI:** 10.3389/fcimb.2022.996841

**Published:** 2022-08-25

**Authors:** Bingyu Jiang, Mingchao Liu, Pei Li, Yue Zhu, Yingying Liu, Kaiqing Zhu, Yuzhu Zuo, Yan Li

**Affiliations:** College of Veterinary Medicine, Hebei Agricultural University, Baoding, China

**Keywords:** lncRNA, ETEC, infection, MPHOSPH9-OT1, CXCL8, IL-8

## Abstract

Enterotoxigenic *Escherichia coli* (ETEC) is a major cause of bacterial diarrhea in piglets, leading to economic losses in the pig industry. In past decades, long non-coding RNAs (lncRNAs) have shown to be widely involved in the regulation of host immunity in porcine infection diseases. In this study, we explored the lncRNAs associated with ETEC F41 infection in IPEC-J2 cells by high-throughput sequencing and bioinformatic analysis. A total of 10150 novel porcine lncRNAs were identified. There were 161 differentially expressed (DE) lncRNAs associated with ETEC F41 infection, of which 65 DE lncRNAs were up-regulated and 96 DE lncRNAs were down-regulated. Functional and KEGG enrichment analysis of predicted target genes of DE lncRNAs indicated they are enriched in cell growth and inflammation-related pathways, such as endocytosis, focal adhesion, TGF-β signaling pathway, and adherens junctions. We revealed a novel candidate lncRNA MPHOSPH9-OT1 that was up-regulated after ETEC infection. The qRT-PCR validation and ELISA assessment showed the knockdown and overexpression of MPHOSPH9-OT1 resulted in significantly down- and up-regulation of cellular mRNA levels and secreted cytokine levels of CXCL8/IL-8, respectively. Meanwhile, MPHOSPH9-OT1 equilibrium is important to maintain the transepithelial electric resistance value and tight junction protein expression of IPEC-J2 cells. This study provides insights into the functionality of novel porcine lncRNAs in host immune responses to ETEC infection.

## Introduction

Enterotoxigenic *Escherichia coli* (ETEC) is the main cause of bacterial diarrhea in piglets, which is widespread in the world and leads to huge economic losses to the pig industry (Do et al., 2006; Luppi, 2017). ETEC colonizes the intestine by binding to host cellular receptors on the small intestinal epithelial cells through the adhesins, which is an important virulence factor including K88 (F4), K99 (F5), F41, 987P (F6), and F18 subtypes (Rajkhowa et al., 2010). Subsequently, ETEC multiplies and forms colonies that cover the surface of villi and begins to release enterotoxins (Acres, 1985). Enterotoxin is a small cysteine-rich peptide that binds to guanylate cyclase located on the surface of intestinal epithelial cells, activating the guanylate cyclase signaling cascade and eventually the cystic fibrosis transmembrane conductance regulator (CFTR), which promotes the secretion of Cl^-^, dehydration of host cells, and finally causes severe diarrhea (Kopic and Geibel, 2010; Mirhoseini et al., 2018).

Many studies reported that ETEC infection triggered innate immune responses of host cells. Tian et al. found that ETEC up-regulated the secretion level of IL-8 in porcine intestinal epithelial cells (IPEC-J2) (Tian et al., 2016). Luo et al. reported that ETEC K88 induced IL-17, IL-21, and IL-23 production in the intestine of the piglets (Luo et al., 2015). Another study indicated that mRNA levels of IL-1β, TNF-α, IL-6, and IL-10 were significantly increased in the jejunum of the piglets after ETEC K88 challenge (Yi et al., 2021). All of the studies lead to the conclusion that the stimulation of host proinflammatory cytokines is mainly regulated *via* the classical TLR or NLRP3 signaling pathways.

In recent years, the involvement of lncRNAs in the regulation of host immunity in porcine viral and bacterial diseases has been reported (Yin et al., 2021; Zhang et al., 2022). LncRNA9606 may regulate intestinal immune IgA production in porcine epidemic diarrhea virus (PEDV)-infected IPEC-J2 cells of neonatal piglets (Chen et al., 2019). LncRNA TCONS00183659 is involved in the expression of inflammatory factors IFIT2, MX1, and MX2, thereby improving the resistance of weaned piglets to ETEC F18 infection (Huang et al., 2019). ETEC F41 is one of the major pathogens causing diarrhea in both piglets and calves, and whether lncRNAs play a novel role in regulating host immune responses post ETEC F41 infection is still unknown. In this study, we explored the lncRNAs associated with ETEC F41 infection in IPEC-J2 cells by high-throughput sequencing and explored the function of a novel candidate lncRNA MPHOSPH9-OT1. Our study reveals the regulatory molecular mechanism of candidate lncRNAs during ETEC infection, and provides a basis for the discovery of new biomarkers or therapeutic targets for piglets diarrhea.

## Results

### ETEC F41 infection of IPEC-J2 cells

**Figure 1:**
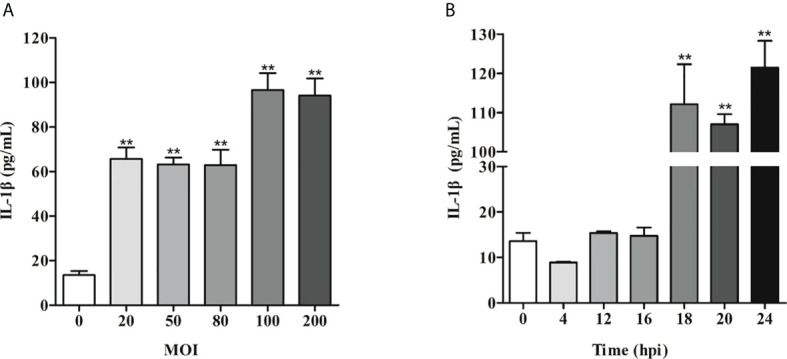
IL-1β induction of IPEC-J2 cells by ETEC infection is MOI- and time-dependent. **(A)** IL-1β secretion induced by ETEC infection at different MOI. **(B)** Time course of IL-1β secretion post-ETEC infection. The x-axis indicates hours post infection. Data are presented as mean ± SEM from 3 independent experiments. **P < 0.01 compared to 0 MOI **(A)**or 0 hpi **(B)**.

To establish an ETEC F41 infected IPEC-J2 cells model, the multiplicity of infection (MOI) gradients (20, 50, 80, 100, 200) were assessed. The ELISA results showed that the IL-1β secretion reaches the highest level at 24 h post infection (hpi) when MOI was 100 (**Figure 1A**). Then IL-1β secretion was measured again to determine the best exposure time of ETEC infection at the MOI of 100. We found the inflammation was significantly induced starting from 18 hpi (**Figure 1B**). Subsequently, the transcriptome of IPEC-J2 cells at 18 hpi with MOI of 100 was analyzed.

### The RNA-seq statistics

A total of 606.18 million clean reads were generated, with an average of 101.03 million reads per sample (**Table 1**). Aligned to the porcine reference genome Sscrofa11.1 (ftp://ftp.ensembl.org/pub/release-94/fasta/sus_scrofa/dna/), on the average of 87.00% and 76.30% of the total clean reads from the wild-type and ETEC-infected cells were able to be uniquely mapped, respectively. While the reads that could not be mapped or mapped to multiple positions were removed. The sequencing quality values of Q20 and Q30 were all above 97% and 93%, with error rates ranging from 0.02% to 0.03%. indicating sequencing results were reliable. Based on fragments perkilobase of exon model per million mapped fragments (FPKM) calculation, we obtained a total of 46,661 and 45,636 transcripts in the wild-type and ETEC-infected IPEC-J2 cells.

**Table 1:** The RNA-seq quality data.

Sample name		Raw reads	Clean reads	Total Mapped reads	Multiple Mapped Reads	Uniquely Mapped Reads	Error rate (%)	Q20 (%)	Q30 (%)
Wild-type IPEC-J2 cells	A	110041084	107036434	103205481 (96.42%)	9952882 (9.3%)	93252599 (87.12%)	0.02	98.51	95.59
	B	93928146	91970500	88262548 (95.97%)	10086616 (10.97%)	78175932 (85%)	0.02	98.42	95.39
	C	107679918	104800844	101125886 (96.49%)	7968877 (7.6%)	93157009 (88.89%)	0.02	98.52	95.48
ETEC-infected IPEC-J2 cells	A	105211202	103652606	94717790 (91.38%)	20257651 (19.54%)	74460139 (71.84%)	0.03	97.28	92.78
	B	107578476	106136726	95904035 (90.36%)	11617474 (10.95%)	84286561 (79.41%)	0.03	97.73	93.68
	C	93881866	92569976	84857237 (91.67%)	12955570 (14%)	71901667 (77.67%)	0.03	97.58	93.43

A, B, C, the relative triplicate in corresponding treatment; Q20, the proportion of bases with a phred base quality score greater than 20; i.e., the proportion of read bases whose error rate is less than 1%; Q30, the proportion of bases with a phred base quality score greater than 30; i.e., the proportion of read bases whose error rate is less than 0.1%.

### The identification of differentially expressed (DE) lncRNAs

**Figure 2:**
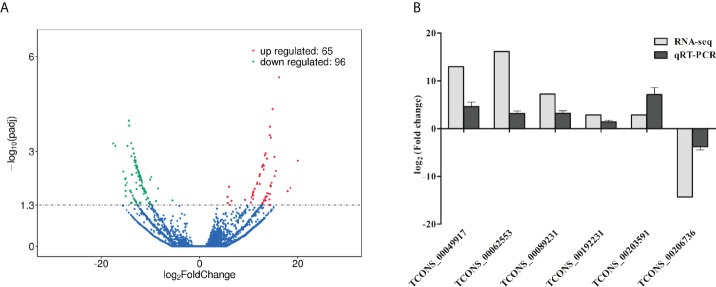
Analysis and validation of DE lncRNAs in ETEC-infected IPEC-J2 cells versus wild-type cells. **(A)** Volcano plot of DE lncRNAs. The x-axis indicates the average values of log_2_ (Fold change), and the y-axis indicates the average values of log_10_ (padj). Comparing the expression levels of ETEC-infected cells versus wild-type cells, the red dots represent the significantly up-regulated lncRNAs (P< 0.05), and the green dots display significantly down-regulated lncRNAs (P< 0.05), and the blue dots indicate the lncRNAs with no statistically difference (P> 0.05). **(B)** Validation of DE lncRNAs using qRT-PCR. The y-axis displays the relative DE lncRNA expression which is represented with log_2_ (Fold change) comparing infected versus wild-type cells. The qRT-PCR analysis was performed using the 2^-ΔΔCt^ method normalized to GAPDH.

A total of 10150 novel porcine lncRNAs were identified in this study. Compared to wild-type cells, 161 DE lncRNAs were detected after ETEC F41 infection, of which 65 DE lncRNAs were up-regulated and 96 DE lncRNAs were down-regulated. The sequencing profile of ETEC-infected IPEC-J2 cells is shown in **Figure 2A**. The top 10 differentially expressed lncRNAs identified in IPEC-J2 cells between ETEC-infection and wild-type, as well as several predicted genes were listed in **Table 2**.

**Table 2:** Top 10 DE lncRNAs between ETEC-infected IPEC-J2 cells and wild-type cells.

lncRNA ID	lncRNA name	Chr	Position (bp)	Gene name	log_2_ (Fold change)	P value	Number of predicted target genes	Examples of predicted target genes
TCONS_00062553	MPHOSPH9-OT1	14	29697947-29728217	MPHOSPH9	16.14291394	3.00E-07	13	RUNDC3B, CDH6, GUCA2B, KANK2, DGKH
TCONS_00198483	LINC4701	8	93059378-93278448	XLOC_130730	15.953249	3.58E-07	12	PDLIM4, TNPO2, SYDE1, GPR52, EPOR
TCONS_00029384	LINC674	11	71700081-72108691	XLOC_020530	16.17182129	4.58E-06	4	CCL3L1, SLC4A8, PLCXD3, ENSSSCG00000037723
TCONS_00159144	SPRYD3-OT6	5	18266504-18298731	SPRYD3	14.87056164	4.62E-05	183	SLC13A5, ZCCHC14, ODF3L2, TENM2, MT3
TCONS_00127720	TGFA-OT2	3	71948146-72054344	TGFA	-14.38167611	0.000106171	6	MYD88, SLC37A3, GPR155, ANKPD34A, RADX
TCONS_00206736	STPG2-AS3	8	122117418-122211921	STPG2	-14.30806276	0.000154252	9	ELOVL2, SS18, C5AR2, CDK2, PDE6C
TCONS_00094545	LINC2200	17	17079071-17210429	XLOC_064739	14.33315253	0.000165002	26	RCOR2, PLCZ1, DRG2, SLC9C1, HASPIN
TCONS_00056533	P2RY12-OT1	13	91378076-91540856	P2RY12	14.31029402	0.000309966	178	SLC13A5, ZCCHC14, ODF3L2, EML6, IL12RB2
TCONS_00003025	LINC87	1	78538954-78724459	XLOC_002225	14.5076655	0.000366392	25	ATP6, LPP, SSH1, TAS2R16, KCNC3
TCONS_00024160	BRINP3-AS1	10	3350813-3522655	BRINP3	-13.89297867	0.000546945	1	BRINP3

Chr, Chromosome in Sus scrofa; Position, LncRNA position on the Sscrofa11.1 porcine genome assembly; Gene name, Genes overlapped with the corresponding LncRNA position on the genome; P value, Represented with adjusted P values; Examples of predicted target genes, listed with some examples of predicted target genes, not all of them.

To validate the RNA-seq results on DE lncRNA screening, six DE lncRNAs were randomly selected to assess their expression levels in ETEC-infected IPEC-J2 cells versus wild-type *via* qRT-PCR. The results showed that the transcript levels of TCONS_00049917, TCONS_00062553, TCONS_00089231, TCONS_00192231, and TCONS_00203591 were significantly up-regulated, while the expression levels of TCONS_00206736 was down-regulated. The trends of the lncRNA expression changes verified by qRT-PCR were consistent with the RNA-seq results, indicating our sequencing results are convincible (**Figure 2B**).

### GO and KEGG enrichment analysis of predicted target genes of host DE lncRNAs

To investigate the roles of host lncRNAs in regulating ETEC infection, we performed Gene Ontology (GO) enrichment analysis on the predicted target genes of 161 DE lncRNAs. **Table 3** listed the top 20 significantly enriched GO categories in ascending order of the P values. They are mainly annotated to intracellular, organelles, lumens, metabolic processes, and protein binding terms. It suggested that lncRNA regulations during ETEC infection are widely involved in diverse cellular components, biological processes, and molecular functions. Particularly, a GO term namely response to biotic stimulus is clearly associated with bacterial infection and host response in the top 20 enriched categories.

**Table 3:** Top 20 significantly enriched GO terms.

GO term	type	P value	Predicted target gene number
nucleus	cellular component	2.35E-17	886
nuclear part	cellular component	7.46E-15	614
intracellular part	cellular component	7.46E-15	1632
membrane-bounded organelle	cellular component	2.72E-14	1304
intracellular membrane-bounded organelle	cellular component	1.36E-13	1295
intracellular	cellular component	1.36E-13	1642
organelle	cellular component	2.52E-13	1436
membrane-enclosed lumen	cellular component	2.64E-13	587
nuclear lumen	cellular component	3.07E-13	554
intracellular organelle	cellular component	5.20E-13	1429
organelle lumen	cellular component	5.35E-13	579
multi-organism process	biological process	5.35E-13	204
intracellular organelle lumen	cellular component	5.94E-13	578
negative regulation of cellular process	biological process	1.12E-12	574
macromolecule metabolic process	biological process	1.40E-12	1136
negative regulation of biological process	biological process	1.47E-12	622
cellular macromolecule metabolic process	biological process	1.47E-12	1040
protein binding	molecular function	1.86E-12	1153
response to biotic stimulus	biological process	8.50E-12	139
binding	molecular function	1.36E-11	1694

P value, Represented with adjusted P values.

**Figure 3:**
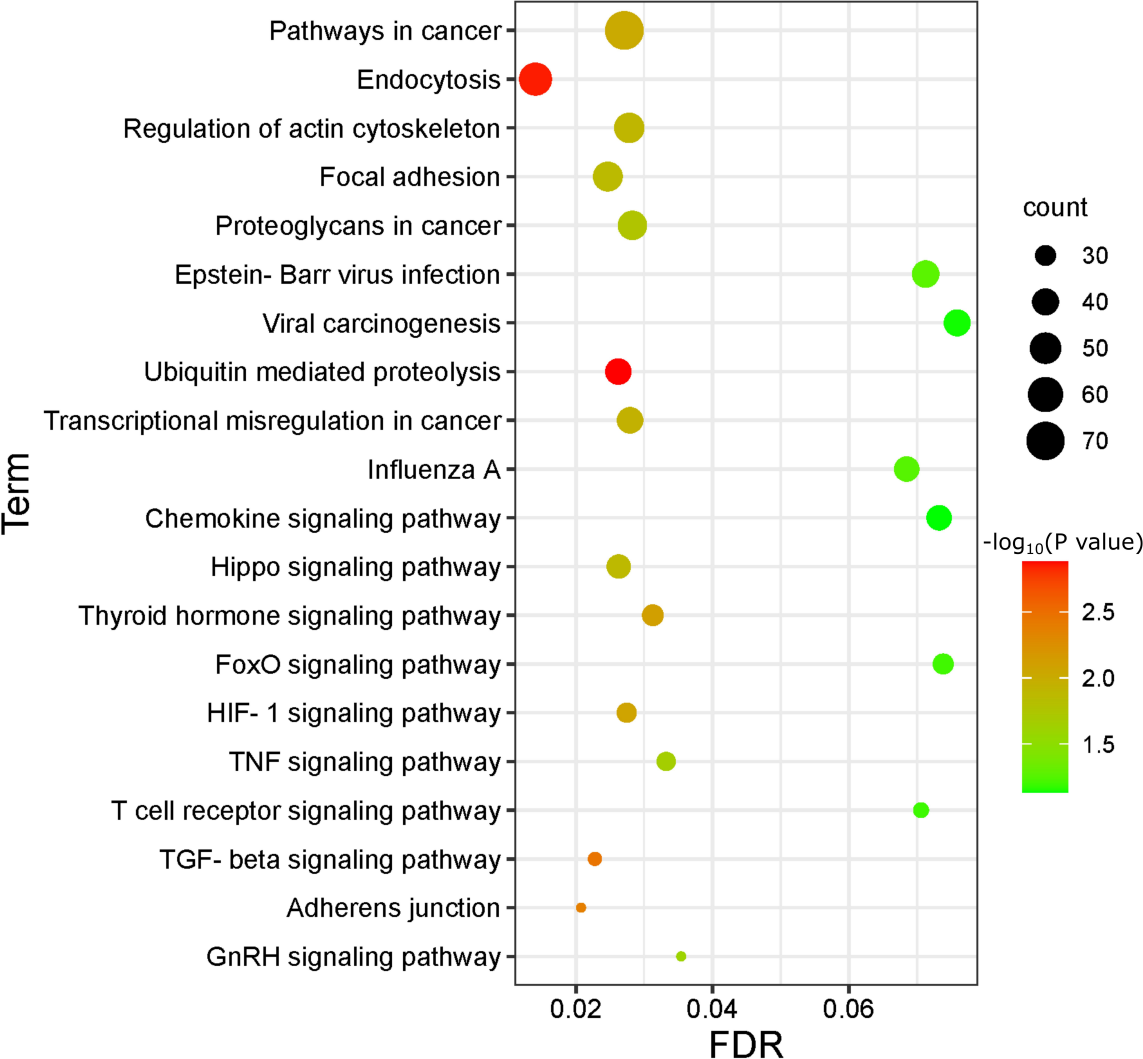
Bubble Plot of KEGG enrichment analysis on predicted target genes of DE lncRNAs comparing ETEC-infected IPEC-J2 cells versus wild-type. The enriched pathways are displayed on the y-axis, and the x-axis represents the false discovery rate (FDR) which means the probability of false positives in all tests. The size and color of dots represent the number of enriched genes and corresponding P-value, respectively.

To discover the relevant host regulatory pathways involved in ETEC infection, Kyoto Encyclopedia of Genes and Genomes (KEGG) enrichment analysis was performed on the predicted target genes of DE lncRNAs. The top 20 significantly enriched pathways were shown in **Figure 3**. The pathways which were related to cell growth and death included pathways in cancer, regulation of actin cytoskeleton regulation, ubiquitin mediated proteolysis, and transcriptional mis-regulation in cancer. The pathways which were associated with infection and host cell responses included endocytosis, focal adhesion, chemokine signaling pathway, Hippo signaling pathway, TNF signaling pathway, TGF-β signaling pathway, and adherens junctions.

### The sequence, structure, and distribution of the novel lncRNA MPHOSPH9-OT1

**Figure 4:**
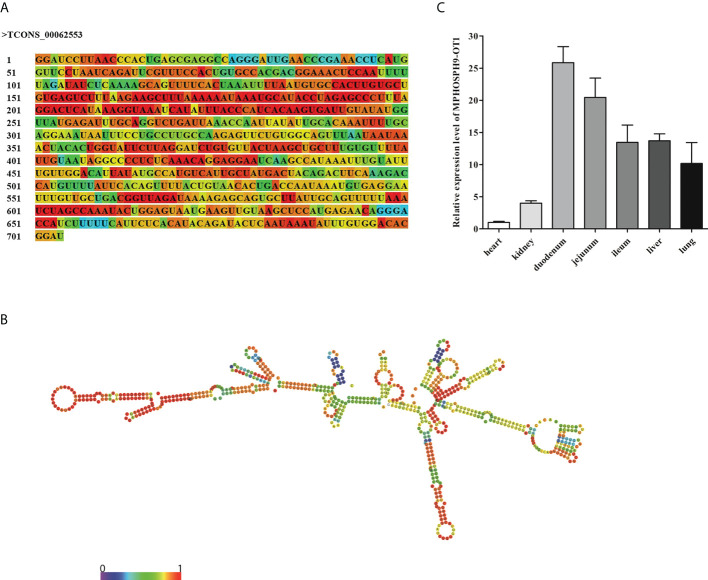
Sequence, structure, and distribution of novel lncRNA MPHOSPH9-OT1. **(A)** The sequence of MPHOSPH9-OT1. The colors of the nucleotides corresponding to the colors in **(B)** indicate the stability of the local secondary structure. **(B)** The predicted optimal secondary structure of MPHOSPH9-OT1 with minimum free energy of -175.10 kcal/mol *via* RNAfold WebServer (http://rna.tbi.univie.ac.at/cgi-bin/RNAWebSuite/RNAfold.cgi). The color from purple to red represents the base-pair probabilities from 0 to 1. A higher probability indicates a stabler base-pairing. **(C)** Distribution and expression of MPHOSPH9-OT1 in porcine organs. LncRNA level of MPHOSPH9-OT1 in each organ was detected using qRT-PCR and quantified by the 2^-ΔΔCt^ method normalized to GAPDH. The y-axis shows the relative expression of MPHOSPH9-OT1 in each organ compared to that in the heart which was set to 1.

The differentially expressed lncRNA TCONS_00062553 is a newly identified lncRNA. It was one of the most significantly differentially expressed lncRNAs after infection with a log_2_ (Fold change) of 16.14 (P<0.01), indicating a critical regulatory role during ETEC infection. The sequence and predicted secondary structure with the minimum free energy of TCONS_00062553 were shown in **Figures 4A, B**. TCONS_00062553 is an intragenic sense strand derived lncRNA with the length of 704 nt, located in the MPHOSPH9 gene cassette on the porcine chromosome 14, therefore we named it MPHOSPH9-OT1. RNA-seq analysis showed no correlation between the expressions of lncRNA MPHOSPH9-OT1 and the gene MPHOSPH9. To investigate the distribution of MPHOSPH9-OT1 in pigs, we examined the expression levels of MPHOSPH9-OT1 in porcine organs by qRT-PCR. The results showed that MPHOSPH9-OT1 was highly expressed in the intestinal tissues, especially in the duodenum and jejunum (**Figure 4C**). Lower transcriptional levels of MPHOSPH9-OT1 in the liver, lung, and kidney were detected, and the lowest transcript level was found in the heart compared to other organs.

### MPHOSPH9-OT1 regulates CXCL8/IL-8 expression during ETEC infection

**Figure 5:**
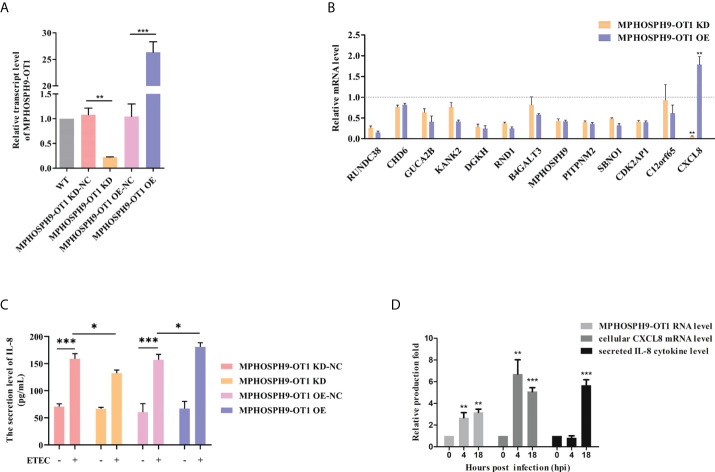
MPHOSPH9-OT1 positively regulates cellular CXCL8 expression and IL-8 secretion after ETEC infection. **(A)** Establishment of MPHOSPH9-OT1 knockdown and overexpression IPEC-J2 cell lines. WT represents wild-type IPEC-J2 cells without any treatments. KD-NC indicates negative control cells transfected with lentiviral vector carrying scrambled sequences for shRNA used in KD cells. OE-NC indicates negative control cells transfected with empty LV5 lentiviral vectors. **(B)** Expression verification of MPHOSPH9-OT1 predicted target genes using qRT-PCR. The qRT-PCR analysis was performed using the 2-ΔΔCt method normalized to GAPDH. The relative transcript levels were calculated using gene expression values of KD/OE cells divided by that of the corresponding negative control cells. The relative transcript values below the grey dotted line mean decreased mRNA levels in given cells compared to corresponding NC, and increased mRNA levels were above the dotted line. **(C)** Assessment of secreted IL-8 levels in the culture medium using ELISA. **(D)** The expression profiles of cellular MPHOSPH9-OT1 transcript, cellular CXCL8 transcript, and secreted IL-8 cytokine levels during ETEC infection. *P<0.05, **P < 0.01, ***P<0.001 compared to 0 hpi.

The MPHOSPH9-OT1 knockdown (KD) and overexpression (OE) IPEC-J2 cell lines were established by lentiviral-mediated delivery of MPHOSPH9-OT1 shRNA or expression vectors. The expression of MPHOSPH9-OT1 in wild-type (WT), KD, KD negative control (KD-NC), OE, and OE negative control (OE-NC) IPEC-J2 cells were confirmed by qRT-PCR (**Figure 5A**). Compared to WT and KD-NC cells, the MPHOSPH9-OT1 transcript level was reduced by more than 70% in KD cells. While MPHOSPH9-OT1 expression was increased more than 26-fold in the OE cells compared with the WT and OE-NC cells.

Thirteen target genes for MPHOSPH9-OT1 were predicted by miRanda (3.3a) and psRobot (v1.2), including RUNDC38, CHD6, GUCA2B, KANK2, DGKH, RND1, B4GALT3, MPHOSPH9, PITPNM2, SBNO1, CDK2AP1, CXCL8, and C12orf65. We detected the mRNA expression levels of the above genes in MPHOSPH9-OT1 KD and OE IPEC-J2 cells. The results showed that in comparison with relative controls, MPHOSPH9-OT1 knockdown decreased CXCL8 mRNA level, while MPHOSPH9-OT1 overexpression promoted CXCL8 mRNA level correspondingly, indicating CXCL8 was the regulatory target gene of MPHOSPH9-OT1 (**Figure 5B**). The decrease and increase of MPHOSPH9-OT1 lncRNA level did not induce corresponding changes in the transcript levels of the other predicted target genes. Since CXCL8 is also named interleukin-8 (IL-8), we further investigated whether MPHOSPH9-OT1 regulates the secretion of IL-8 cytokine levels during ETEC infection (**Figure 5C**). ETEC infection stimulated IL-8 secretion of control IPEC-J2 cells. However, ETEC infection of MPHOSPH9-OT1 KD cells resulted in significantly lower IL-8 secretion than that of the infected control cells (KD-NC). Coherently, the overexpression of MPHOSPH9-OT1 in ETEC-infected IPEC-J2 cells (OE) significantly elevated the secreted IL-8 levels compared to infected control cells (OE-NC). We next characterized the expression profile of intracellular CXCL8 mRNA and extracellular IL-8 cytokine at different time points during ETEC infection. **Figure 5D** shows cellular MPHOSPH9-OT1 and CXCL8 mRNA level was immediately up-regulated 4 h post ETEC infection and stayed at the high expression level until 18 h, while IL-8 secretion was not able to be detected until 18 h post infection. In summary, MPHOSPH9-OT1 probably up-regulates CXCL8/IL-8 expression but not secretion during ETEC infection.

### The dysregulation of MPHOSPH9-OT1 disrupts the epithelial barrier function of IPEC-J2 cells

**Figure 6:**
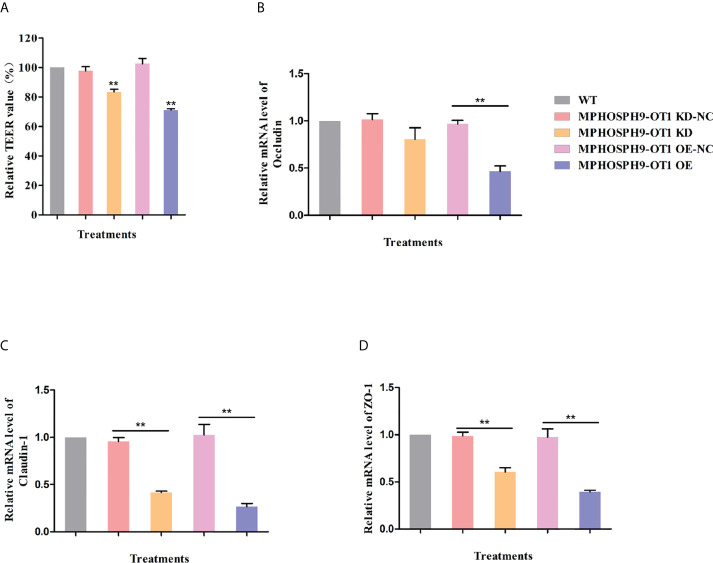
MPHOSPH9-OT1 knockdown or overexpression negatively affects the barrier function of IEPC-J2 cells. **(A)** The KD and OE of MPHOSPH9-OT1 down-regulated TEER values of monolayer IPEC-J2 cells. **P < 0.01 compared to WT. **(B–D)**The KD and OE of MPHOSPH9-OT1 decreased the transcript level of Occludine **(B)**, Claudin-1 **(C)**, and ZO-1 **(D)**. The expression analysis was performed using qRT-PCR and calculated by the 2^-ΔΔCt^ method normalized to GAPDH. The relative expression levels of all treatments were shown with the expression fold number compared to WT. **P < 0.01 .

Since it has been demonstrated that ETEC infection disrupts intestinal barrier function, we detected the role of candidate lncRNA MPHOSPH9-OT1 in maintaining transepithelial electrical resistance (TEER) in IPEC-J2 cell monolayers. As shown in **Figure 6A**, both knockdown and overexpression of MPHOSPH9-OT1 resulted in significantly lower TEER values in IPEC-J2 cell monolayers. Moreover, we also examined the effect of MPHOSPH9-OT1 on the expression of tight junction proteins. Compared to WT and control IPEC-J2 cells, both the knockdown or overexpression of MPHOSPH9-OT1 down-regulated Occludin, Claudin-1, and ZO-1 mRNA levels significantly or not significantly, indicating a disruption of intercellular tight junctions and intestinal barrier integrity (**Figures 6B–D**). Interestingly, in comparison to MPHOSPH9-OT1 knockdown, its overexpression resulted in more severe damage to the TEER and the tight junction of IPEC-J2 cells. In conclusion, the dysregulation of lncRNA MPHOSPH9-OT1 level increased the permeability of intestinal cells and disrupted the intestinal barrier function.

## Discussion

In the present study, we showed that ETEC infection induced expression changes of various lncRNAs in IPEC-J2 cells. These differentially expressed lncRNAs are widely involved in cell integrity, cell proliferation, apoptosis, and immune-related biological processes and molecular pathways, indicating that lncRNAs play an important regulatory role in the process of ETEC infection. We identified a novel lncRNA MPHOSPH9-OT1 that may affect intestinal barrier function by regulating the expression of CXCL8. This study provides a theoretical basis for further elucidating the role of host lncRNAs on ETEC infection regulation and offers novel biomarkers for ETEC-induced diarrhea in piglets.

In this study, we established an ETEC-infected IPEC-J2 cell model based on the secretion of IL-1β. In this condition, the predicted target genes of differentially expressed lncRNAs were significantly enriched in membrane structural functions such as membrane-bounded organelle, membrane-enclosed lumen, and organelle lumen, which together maintain cellular homeostasis. While the enterotoxin secreted by ETEC can activate CFTR-regulated chloride channels, leading to the secretion of Cl^-^ and inhibition of NaCl absorption, resulting in loss of electrolytes and water, which eventually causes diarrhea by damaging cell homeostasis (Berberov et al., 2004). We propose DE lncRNAs may be involved in diarrheal symptoms by modulating membrane homeostasis. Particularly, we found that GUCA2B, the common target gene of DE lncRNAs TCONS_00062553, TCONS_00080642, TCONS_00216349, TCONS_00125414, TCONS_00037594, TCONS_00231859, and TCONS_00237113, is an upstream regulator of the CFTR pathway with the function of regulating water and electrolyte homeostasis. Therefore, the function of these lncRNAs may mediate porcine small intestinal epithelial cell homeostasis during ETEC infection, which needs further investigation.

Several studies have reported the involvement of host lncRNAs in regulating the host cell inflammatory responses during bacterial infection. The dysregulated immune related genes ABCB1, STAT1, and CLEC7A could be regulated by lncRNA XLOC_078370 in the Ileum of Piglets infected with Clostridium perfringens Type C (Huang et al., 2019). LncRNA XIST antagonizes NF‐κB/NLRP3 pathway to regulate inflammation of bovine mammary alveolar cells infected with *E. coli* and *Staphylococcus aureus* (*S. aureus*) (Augustino et al., 2020). Most previous studies focused on the production of host pro-inflammatory factors induced by ETEC infection such as IL-18, IL-1β, IL-6, IL-1α, and TNFα (Kern et al., 2017; Lin et al., 2021). Interestingly we identified a large number of target genes of lncRNAs like TCONS_00147108、TCONS_00078061、TCONS_00003410、TCONS_00213513、TCONS_00005258、TCONS_00096374 were found to be enriched in the TGF-β signaling pathway. TGF-β is an anti-inflammatory cytokine that plays important role in mucosal defense, wound healing, immune tolerance, and many diseases like inflammatory bowel diseases (IBD) and cancers. Bahrami et al. reported that TGF-β can be stimulated by ETEC infection in human intestinal epithelial cell lines (Bahrami et al., 2011). On the contrary, Xu et al. revealed a significant decrease in serum TGF-β levels in piglets challenged with ETEC K88ac. The regulatory function of host TGF-β in ETEC infection remains controversial. Our results indicate the above candidate lncRNAs might potentially interact with TGF-β to mediate the inflammatory response in the host gut during bacterial infection. Future studies on these candidate lncRNAs could help to elucidate the regulatory mechanism of TGF-β signaling pathway during ETEC-induced diarrhea.

In recent years, many studies have found that lncRNAs are involved in the regulation of mucosal barrier function in a variety of gastrointestinal diseases, by affecting the expression of tight junction proteins. Xiao et al. reported that lncRNA SPRY4-IT1 affects intestinal epithelial barrier function in mice exposed to septic stress by regulating the stability and translation of tight junction mRNAs (Ray et al., 2004). LncRNA CDKN2B-AS1 plays a negative role in colonic epithelial barrier function by downregulating Claudin-2 expression in IBD patients (Triner et al., 2018). Myc-associated zinc finger protein (MAZ) is a Cys2-His2 type zinc finger transcription factor that positively controls the transcription of tight junction proteins ZO-1 and occludin in IBD patients (Chen et al., 2017). In this study, candidate lncRNAs TCONS_00082795, TCONS_00047114, TCONS_00139491, TCONS_00178088, TCONS_00106149, TCONS_00075100, TCONS_00172176, TCONS_ 00067616, TCONS_00213212, all have MAZ as one of the predicted target genes, thus we speculate that they might be involved in modulating the mucosal barrier dysfunction caused by ETEC infection, which needs to be further investigated.

In this study, we verified CXCL8 as a target gene of lncRNA MPHOSPH9-OT1. Overexpression of MPHOSPH9-OT1 significantly up-regulated CXCL8 mRNA level, while knockdown of MPHOSPH9-OT1 significantly decreased CXCL8 mRNA level in IPEC-J2 cells. CXCL8, also known as interleukin-8 (IL-8), belongs to the CXC subfamily of chemokines that mainly participate in maintaining cellular homeostasis and regulating inflammatory responses (Russo et al., 2014). CXCL8 also plays a predominant role in the pathogenesis of many intestinal inflammatory diseases including Ulcerative colitis and IBD (Daig et al., 1996; Zhu et al., 2021). Meanwhile, elevated CXCL8 secretion was detected both in the serum and the small intestinal mucosa of the diarrheic mouse model caused by intestinal dysbiosis (Hansen et al., 2013). It was reported that IPEC-J2 cells secret IL-8 for host immune responses against ETEC K88ac infection (Tian et al., 2016). Together with our finding, lncRNA MPHOSPH9-OT1 might be a key upstream mediator for the stimulation of CXCL8/IL-8 production in IPEC-J2 cells infected with ETEC.

There is increasing recognition of the relationship between epithelial inflammation and gut barrier function. Treating human colonic epithelial cells (T84 cells) with IL-4 or IL-13 resulted in a significant decrease in TEER and an increase in epithelial permeability (Ceponis et al., 2000), indicating a direct association with pro-inflammatory cytokines with the epithelial barrier function. The mucosal barrier is a layer of epithelial cells that physically separates the intestinal lumen and the internal milieu. The intestinal immune reaction contributes to the mucosal barrier function as a defense against pathogens. It has been shown that the probiotic *Enterococcus faecium* HDRsEf1 (Ef1) could inhibit the production of IL-8 of IPEC-J2 cells infected with K88ac, at the same time increasing the TEER, indicating epithelial IL-8 is negatively correlated with mucosal barrier function (Tian et al., 2016). A study by Sansonetti et al. further confirmed that IL-8 promotes epithelial barrier destruction and bacterial invasion during shigellosis (Sansonetti et al., 1999). Nevertheless, IL-8 was shown to be a key mediator for human intestine development that protects the mucosal barrier against chemical injury, implicating IL-8 is positively correlated with gut barrier function (Maheshwari et al., 2002). Apparently previous interpretations of IL-8 regulation in epithelial barrier functionality remain controversial. We figured that a stable expression of novel lncRNA MPHOSPH9-OT1 which targets and regulates IL-8 mRNA level is critical to the barrier function of IPEC-J2 cells. Either up-or down-regulated MPHOSPH9-OT1 RNA level, the value of TEER, and the expression of tight junction proteins (Claudin-1 and ZO-1) all significantly decreased. Our results explained one possibility that MPHOSPH9-OT1 equilibrium which controls IL-8 cytokine at an appropriate cellular concentration might be essential for epithelial barrier function. Except for MPHOSPHO9-OT1, an integrative bioinformatic analysis on lncRNAs and mRNA in piglet ileum infected with *Clostridium perfringens* Type C found several candidate lncRNAs had CXCL8 as predicted target genes, ncluding XLOC_078370, ALDBSSCG0000005727, ENSSSCG00000015579, XLOC_007804, and XLOC_020704 (Huang et al., 2019).

In conclusion, we identified 161 differentially expressed lncRNAs in IPEC-J2 cells associated with ETEC F41 infection, of which 65 DE lncRNAs were up-regulated and 96 DE lncRNAs were down-regulated. Novel lncRNA MPHOSPH9-OT1 regulated the expression of target gene CXCL8/IL-8, and affected the intestinal barrier function. Our study provides insights into the function of host lncRNAs during ETEC infection.

## Materials and methods

### Cell culture and ETEC infection

The IPEC-J2 cells were purchased from Guangzhou Jennio Biological Company (Guangzhou, China). ETEC F41 Strain was purchased from the Chinese Veterinary Culture Collection Center. IPEC-J2 cells were cultured in Dulbecco’s Modified Eagle’s medium (DMEM) containing 10% fetal bovine serum (FBS) (Gibco, USA) at 37°C in a 5% CO_2_ incubator until reaching 90% confluence. The medium was replaced with Eagle’s Minimal Essential Medium (MEM) (Gibco, USA), and infected with the ETEC F41 strain with an MOI of 100. After infection at 37°C and 5% CO_2_ for 1 h, the cells were rinsed with sterile PBS 3 times to remove residual bacteria, and finally cultured in DMEM containing 10% FBS. After incubation for another 17 hours, the infected cells were collected as ETEC-infected samples (18 hpi). Uninfected IPEC-J2 cells were collected as wild-type control (0 hpi). Each treatment was performed in triplicates.

### RNA isolation, library construction, and RNA-seq

Total RNA was extracted from IPEC-J2 cells according to TRIzol (Invitrogen, USA) reagent instructions. RNA was tested for degradation and contamination by a 1% agarose gel. RNA purity was measured using the NanoPhotometer^®^ spectrophotometer (IMPLEN, USA). The Qubit^®^ RNA Assay Kit (Life Technologies, USA) was utilized to measure the RNA concentration, and the RNA Nano 6000 Assay Kit (Agilent Technologies, USA) was used to detect the RNA integrity.

RNA samples were sent to Beijing Novogene Co. Ltd. for library construction and sequencing. RibozeroTM rRNA Removal Kit (Epicenter, USA) was used to remove ribosomal RNA. The linear RNA was digested with RNase R (Epicentre, USA), followed by using the NEBNext^®^ UltraTM RNA Library Prep Kit (NEB, USA) to construct the cDNA libraries. cDNAs of about 250-300 bp length were screened using the AMPure XP system (Beverly, USA). Finally, the library quality was evaluated by Agilent Bioanalyzer 2100 system and sequenced with the Illumina PE150 platform.

### Sequencing data assessment and analysis

The clean reads were filtered by removing the adaptors, raw reads with unknown bases, and low-quality reads. To check the sequencing quality, Q20, Q30, and GC content were assessed. The clean reads were mapped to the reference genome using Tophat (V2.0.9) to filter out known and novel transcripts. Novel transcripts with a length greater than 200 bp were selected for coding ability prediction using CPC software (0.9-r2), and novel lncRNAs were screened by filtering out transcripts with the coding ability and unclassified transcripts.

### DE lncRNA analysis and target gene prediction

Cuffdiff (2.1.1) and edgeR (3.2.4) were used to analyze DE lncRNAs between the two treatments. Benjamini & Hochberg method was used to adjust the P-value. When the corrected P-value (Padj) < 0.05, and the absolute value of the expression change fold ≥2, the expression difference of the lncRNAs between normal and ETEC infected IPEC-J2 cells was considered significant.

We used miRanda (3.3a) and psRobot (v1.2) to predict the target gene, through the genome position and expression correlation of lncRNAs and protein-coding genes, respectively. The threshold for position analysis was set at 100 kb upstream and downstream from the position of the lncRNA. Subsequently, GOSeq Release 2.12 and KO-BAS (V2.0) were utilized to perform GO and KEGG pathway enrichment analysis of the DE lncRNAs, respectively.

### qRT-PCR

Based on the sequencing results, five differentially expressed lncRNAs were randomly selected for expression validation. The primers were designed using the Primer-BLAST function on the NCBI website (https://www.ncbi.nlm.nih.gov/tools/primer-blast/) (**Table 4**). Reverse transcription was performed with HiScript II 1st Strand cDNA Synthesis Kit (Vazyme, Nanjing, China) and qRT-PCR was performed using SYBR Green Supermix kit (Bio-Rad, USA). The relative expression levels of lncRNA were analyzed using the 2^-ΔΔCt^ method normalized to GAPDH.

**Table 4:** Primer sequences used in qRT-PCR.

Transcript ID/Gene Symbol	FW sequence	RV sequence
TCONS_00049917	AACAGCATACTGACAAGCAGA	AGAAGTGCGCCAAATAGAGC
TCONS_00062553	ATTTCCTGCCTTGCCAAGAGT	CTCCTGTTTGAGAGGGGCCTA
TCONS_00089231	CAACCCATTTTGGTGGGGAG	TGCTGATTTCAAGTGAAGCCT
TCONS_00192231	GAGGAGCTCTTTTGAGGAGAGAG	TGATTCACTGAGACCCAGAGC
TCONS_00203591	AGGTTCCCACAACCAACCTG	ACTGCCATTTAGTTGACATCTG
TCONS_00206736	TTAACGAATCCGACTAGGAA	CCAGGCTAGGGGTTGAATCG
AATCGGAPDH	AACGTGTCGGTTGTGGATCT	TCACAGGACACAACCTGGTC
occludin	CTCCGGGGAGAGCTAGAC	TGGGTGCATAATGATTGGGT
claudin-1	CAACACCAAGGCCCTATCCA	CACATGAAAATGGCTTCCCTCC
ZO-1	CAACAGCATCCTCCCACCTT	TCACAGTGTGGTAAGCGCA
CXCL8	TGCAGAACTTCGATGCCAGT	AATTCTTGGGAGCCACGGAG

FW sequence, Forward primer sequences; RV sequence, Reverse primer sequences.

### Collection of porcine tissue samples

A total of six Large White piglets which were raised under the same husbandry condition were euthanized by exsanguination under anesthesia at the age of 28 days. Tissue samples were collected from each piglet, including heart, kidney, duodenum, jejunum, ileum, liver, and lung were collected. All samples were rapidly frozen in liquid nitrogen followed by storage at –80 °C.

### Construction of MPHOSPH9-OT1 knockdown and overexpression of IPEC-J2 cells

A lentivirus-mediated RNAi technology was applied to knock down lncRNA MPHOSPH9-OT1 in IPEC-J2 cells. The shRNA sequence for MPHOSPH9-OT1 (GGAAATAATTTCCTGCCTT) and a scrambled sequence (TTCTCCGAACGTGTCACGT) were used to construct the lentiviral vectors (LV3) that carry GFP for the establishment of MPHOSPH9-OT1 KD cells and KD-NC cells, respectively. The construction was performed by the GenePharma Company (Suzhou, China). Subsequently, the IPEC-J2 cells were infected with the above lentiviral vectors. GFP was observed 24 h post lentivirus infection to evaluate the infection efficiency by inverted fluorescence microscope (ZEISS, Germany). To select the GFP-positive cells, 10 μg/mL puromycin was added to the cell culture 72 h post lentivirus infection. The surviving cells were assessed for MPHOSPH9-OT transcript levels *via* qRT-PCR.

The recombinant MPHOSPH9-OT1-overexpression and corresponding negative control lentiviral vectors (LV5) were customized by the GenePharma Company (Suzhou, China). To establish MPHOSPH9-OT1 OE cells and OE-NC cells, the subsequent lentiviral infection, GFP screening, puromycin selection, and expression assessment by qRT-PCR were performed with the methods described above.

### TEER measurement

The wild-type IPEC-J2 cells, MPHOSPH9-1KD cells and MPHOSPH9-1 OE cells were inoculated into the upper chamber of Transwell plates (Corning, USA), respectively. When the cells form a monolayer cell membrane structure, the TEER values were measured using Millicell ERS-2 voltohmmeter (Millipore, USA).

### Statistical analysis

Statistical significance was determined using two independent samples t-test *via* SPSS (19.0) software. A probability of P< 0.05 was considered statistically significant. 0.01 < P < 0.05 was indicated by *, P < 0.01 was indicated by**, P<0.001 was indicated by ***.

## Data availability statement

The datasets presented in this study can be found in online repositories. The names of the repository/repositories and accession number(s) can be found below: https://www.ncbi.nlm.nih.gov/, PRJNA854045.

## Ethics statement

The animal study was reviewed and approved by Animal Ethical and Welfare Committee (AEWC) of Hebei Agricultural University.

## Author contributions

Conceptualization and methodology, YZZ and YL; Experimental investigation and data analysis, BJ, ML, PL, YZ, YYL, and KZ; data curation, BJ, ML, and PL; writing—original draft preparation, BJ, ML, and PL; writing—review and editing, YZZ and YL; supervision, ML, YZZ, and YL; project administration and data acquisition, YL. All authors have read and agreed to the published version of the manuscript.

## Funding

This research was funded by the Natural Science Foundation of Hebei Province (Grant number 2020204072) and the China Postdoctoral Science Foundation (2019M661954).

## Acknowledgments

We would like to acknowledge Dr. Wanzhe Yuan at Hebei Agricultural University for his help with porcine tissue sample collection.

## Conflict of interest

The authors declare that the research was conducted in the absence of any commercial or financial relationships that could be construed as a potential conflict of interest.

## Publisher’s note

All claims expressed in this article are solely those of the authors and do not necessarily represent those of their affiliated organizations, or those of the publisher, the editors and the reviewers. Any product that may be evaluated in this article, or claim that may be made by its manufacturer, is not guaranteed or endorsed by the publisher.
